# Old Practitioners

**Published:** 1906-05

**Authors:** Gustavus North

**Affiliations:** Cedar Rapids, Iowa.


### OLD PRACTITIONERS.

By Dr. Gustavus North, Cedar Rapids, Iowa.

Great advancement has been made the past few years, in all branches of the dental profession. A successful practitioner must be up and pushing forward.

The public demand first class operators, and they soon find out the progressive and successful man.

As my essay is especially for old practitioners, the young men will find but little food in my remarks. If the old practitioner does not keep pace with the times he will soon be left behind.

I have lately talked with a number of old practitioners in regard to many of the new features now before the profession, and they seem to take but little interest in the advancement, and perhaps answer by saying "I practiced dentistry many years before these young fellows were born." That may be true, but some of these young men are model men, and are worthy to follow, they can teach some older members the true arts of dentistry. I know some young men that are skillful operators, the best I ever saw. I am proud of the up-to-date young practitioners, they are worthy of their calling.

Some of the older men in the profession are practicing the old methods of thirty, and even forty years ago, hammering along in the same old rut. I wish to mention just one feature in operative dentistry in this short article, and that is the old and the new way of preparing cavities in the proximal surfaces of bicuspids and molars. Many years ago we prepared such cavities in a dovetail shape with deep under cuts on each side of the the cavity, leaving the walls thin and frail, with a dish-shaped base. In a very short time the thin enamel walls would crumble away, and the tooth would be in a bad condition.

But listen, old practitioners, just reverse the shape of the cavities, make them broad and flaring with strong enamel walls, with a square base, and a step anchorage, and you will have an ideal cavity, and when properly filled will stand for years. You will be surprised to see the difference in the preservation of the teeth. Remember and make good round contact surfaces, don't forget and make the old flat surfaces or wide spaces between the fillings, but have your contact points meet and you will be pleased with your efforts.

Take advice, old practitioners, from a friend that is in his fortieth year of constant practice. I am making better fillings today than ever before. I can stand at my chair from morning until night with better grace knowing that I am not a back number, but keeping pace with the times. I intend to be a boy with the boys as long as I practice.

